# Reduced Fatigue Symptoms in the Post-COVID Syndrome With Amifampridine: A Collective Casuistry With Double-Blind Discontinuation Trials

**DOI:** 10.7759/cureus.52935

**Published:** 2024-01-25

**Authors:** Thomas Boehmeke

**Affiliations:** 1 Cardiology, Cardiology Gladbeck Germany, Gelsenkirchen, DEU

**Keywords:** bell score, amifampridine, fatigue, sars-cov-2, post-covid syndrome

## Abstract

After a severe acute respiratory syndrome coronavirus 2 (SARS-CoV-2) infection, approximately 10-20% of patients are affected by the post-COVID syndrome (PCS). This condition leads to a variety of functional complaints, including symptoms of fatigue. To date, there is still no adequate treatment option. Five patients are presented, including the self-observation of one of the authors, in whom the administration of amifampridine as an "off-label use" led to a normalization of the unphysiologically increased need for sleep with a simultaneous increase in the Bell score. This effect was confirmed in a double-blind discontinuation trial (the medication was discontinued on a trial basis) in two of the patients. The five patients, who were previously unable to lead a normal life due to their fatigue symptoms, were able to return to everyday life after treatment with amifampridine. This offers hope to millions of affected patients.

## Introduction

The prevalence of post-COVID syndrome (PCS) is 10-20% following a SARS-CoV-2 infection. In addition to specific organ diseases, a variety of functional complaints occur, primarily symptoms of fatigue, secondary shortness of breath, chest pain, and disorders in smell, taste, memory, and concentration, as well as palpitations and a lack of resilience [1-3]. The proportion of patients with individual symptoms is significantly higher [4, 5]. Despite the numerous guidelines available, there are no established treatment methods for PCS [6, 7]. Most medications that have been tried have proven unsuitable [8].

By December 2023, there were a total of around 700 million confirmed cases of COVID-19 worldwide [9]. Combining this figure with the prevalence, the global number of PCS cases is likely to be in the high double-digit millions. Given these high numbers of PCS cases, there is significant interest in finding suitable substances to improve PCS-associated symptoms.

Many important discoveries in medicine have been made by chance. Probably the best-known examples are penicillin and sildenafil. The author (T.B.) has been suffering from myasthenic syndrome since 2021. In May 2022, T.B. contracted SARS-CoV-2, followed by severe fatigue with an extensive need for sleep. In August 2022, treatment with amifampridine (3,4-diaminopyridine) was initiated on suspicion of Lambert-Eaton myasthenic syndrome (LEMS). Immediately after the first dose of amifampridine, not only the muscular deficits but also the severe fatigue symptoms improved. Several of T.B.'s relatives also suffered from PCS with severe fatigue and impaired cognition but without neurological pre-existing conditions. At their request, individual treatment trials were conducted as "off-label use" of amifampridine. These patients also experienced a marked improvement in their fatigue symptoms.

## Case presentation

T.B. (patient 1) provided his relatives (patients 2 to 5) with comprehensive and detailed information, and extensive literature on amifampridine was made available to them. Patients 2 to 5 gave written informed consent for the individual treatment trials with amifampridine. In accordance with the guidelines of the ethics committees, other etiologies and potential contraindications were excluded. Patients 2 to 5 were refractory to other therapy attempts, such as physiotherapy and occupational therapy, to improve fatigue.

The Bell score was used for evaluation, focusing on the restriction of vigilance, physical resilience, occupational activity, and the cumulative sleep duration over 24 hours. To evaluate potential placebo effects, a double-blind discontinuation trial (3 days verum, 3 days placebo) was conducted at the request of patients 2 and 3, who were on long-term amifampridine medication.

The verum and placebo capsules were prepared by a wholesale pharmacy; the blinding was carried out by a colleague. The Bell score and the need for sleep per 24 hours were documented daily by T.B.

Patient 1, who was a 64-year-old individual, has had a neuromuscular disease since 2021, which was clinically classified as myasthenia gravis due to seronegativity. No significant improvement could be achieved with the appropriate drug treatment. After a SARS-CoV-2 infection in April 2022, severe fatigue occurred, with sleep time being 16-18 hours/24 hours, and the Bell score was 10. Due to the refractory nature of the standard therapies for the suspected myasthenia gravis, treatment with amifampridine was initiated in August 2022 under differential diagnostic aspects. The fatigue ceased, and the sleep time was reduced to 8-9 hours/24 hours. With improved muscular resilience, the Bell score increased to 80, and the ability to work was regained. A clinical diagnosis of Lambert-Eaton myasthenia syndrome was thus made.

After a SARS-CoV-2 infection in April 2022, 63-year-old patient 2 experienced fluctuating fatigue symptoms with a Bell score between 20 and 40 and an increased need for sleep at 12 hours per 24 hours. After taking 5 mg amifampridine from October 2022, there was complete convalescence with a Bell score of 100; sleep time was reduced to 7 hours/24h. After discontinuation trials, recurrent fatigue occurred after a few days, following which long-term medication with a daily dose of 20 mg was established. Cognition was restored, and physical resilience normalized.

After two SARS-CoV-2 infections in January 2021, 26-year-old patient 3 had a reduced ability to concentrate, her sleep duration was 11-12 hours / 24 h and her Bell score was 40. It was hardly possible to continue her medical studies under these conditions. After taking amifampridine from May 2022, the need for sleep fell to 7-9 hours and the Bell score increased to 80. The effect lasted about 36 hours and was reproducible by taking amifampridine again. A thorough stabilization was achieved with long-term medication at a daily dose of 20 mg. Medical studies could be continued without any relevant restrictions.

After SARS-CoV-2 infection in January 2022, 61-year-old patient 4 developed severe fatigue symptoms, the Bell score was 40 and the sleep time increased to 12 hours per 24 hours. Vigilance was significantly impaired during the waking phases, which led to a considerable restriction of occupational activity. After 5 mg amifampridine from October 2022, the Bell score increased to 100 and the sleep time fell to 7.5 hours /24 h. Vigilance improved completely. Subsequently, 5 mg amifampridine was taken on an as-needed basis, with no symptoms.

**Figure 1:**
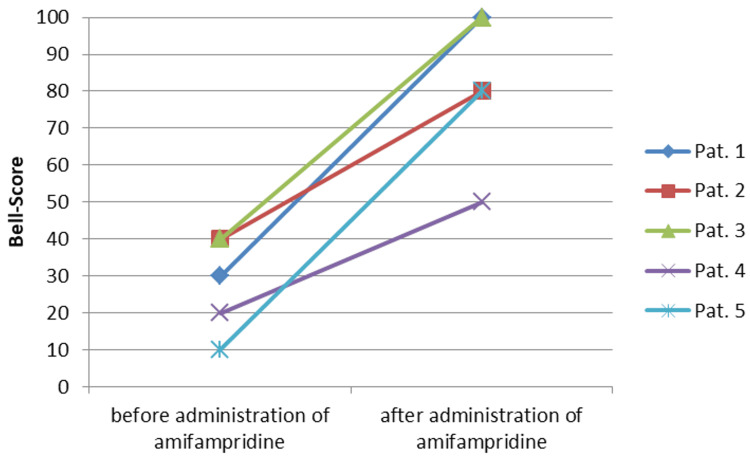
Development of the Bell score of the five patients after administration of amifampridine

**Figure 2:**
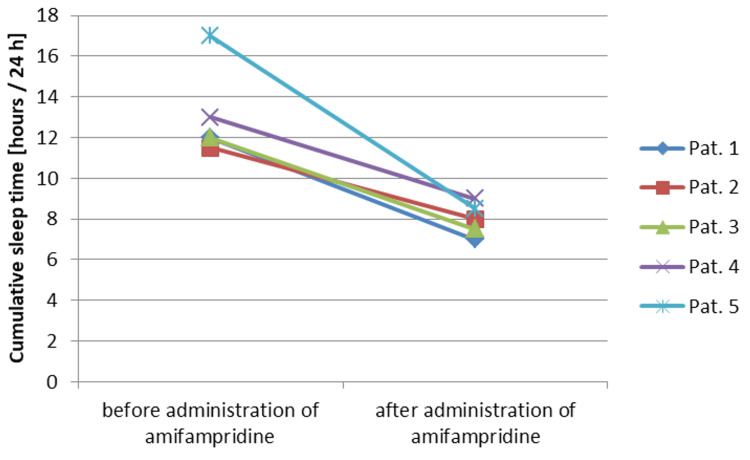
Development of the cumulative sleep time of the five patients after administration of amifampridine

During the first wave of COVID-19, 36-year-old patient 5 was caring for severely ill patients in the intensive care unit, shortly after which the typical symptoms of a COVID-19 infection with loss of taste occurred in spring 2020. Post-Covid myocarditis was diagnosed with significantly reduced physical resilience. The Bell score at this time was 20 and the sleep time was increased to 12-14 hours per 24 hours. A therapy trial with 5 mg amifampridine was carried out 1.5 years after the onset of symptoms. Within one day, the patient experienced a reduction in fatigue symptoms and muscular complaints (Bell score 50) (Figure 1). The sleep time was reduced to 8 to 10 hours per 24 hours (Figure 2). The positive effects on exercise tolerance and sleep time were reproducible by taking 5 mg amifampridine as needed. The remaining limitation of exercise capacity is due to cardiac-associated problems; after cardiac rehabilitation, a return to work is planned.

In summary, the Bell score increased significantly in all five patients (Figure 1). The increase from a mean of 28 ± 16 to 82 ± 20 was highly significant (p=0.00265, paired t-test).

In patients 2 and 3, long-term medication with 20 mg amifampridine per day was required for convalescence, thus providing the prerequisite for a double-blind discontinuation trial. This was carried out over six days in June 2023. The discontinuation trial began eight months after the start of treatment for patient 2 and two months after the start of treatment for patient 3.

**Figure 3:**
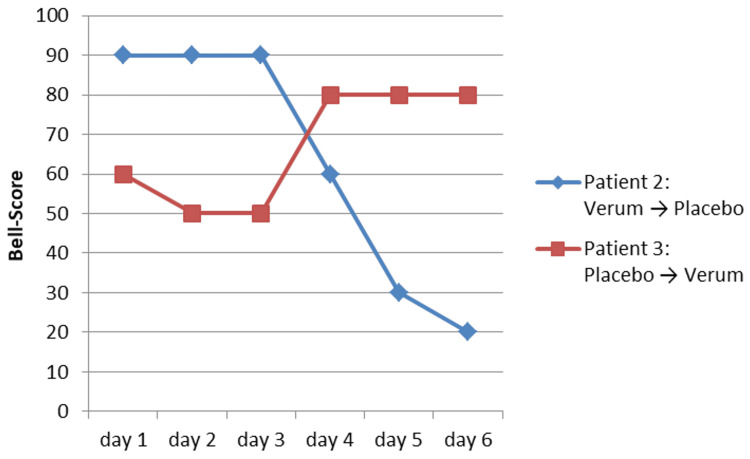
Results of the discontinuation trial for the Bell score

**Figure 4:**
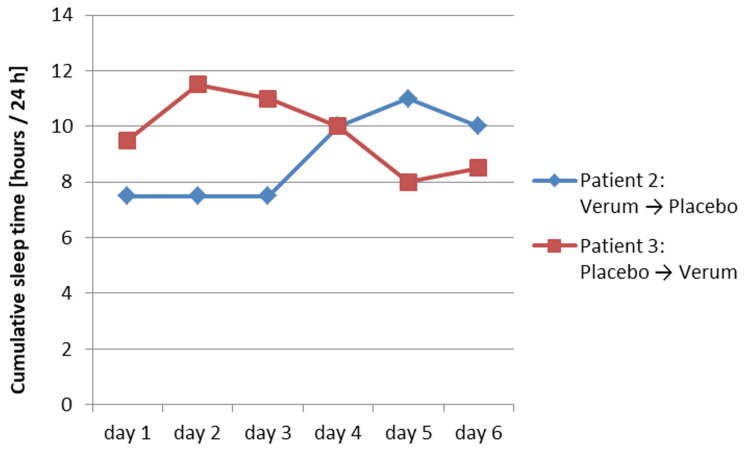
Results of the discontinuation trial for the sleep time

In patient 2, there was a strong reduction in the Bell score from 90 to 60 (day 4) to 20 (day 6) after discontinuation (placebo) from day 4 (Figure 3). The sleep time increased from 7.5 to 10.0 to 11.0 hours (Figure 4).

In patient 3, the discontinuation (placebo) led to a reduction in the Bell score to 50-60 from day 1 (Figure 3). The sleep time increased to up to 11.5 hours (day 2). After termination of the discontinuation (verum) from day 4, the Bell score normalized to 80 and the sleep time decreased again to 8.0 hours (day 5) (Figure 4).

## Discussion

The author (T.B., patient 1), who suffers from Lambert-Eaton myasthenic syndrome (LEMS) in combination with post-COVID syndrome (PCS), observed an improvement in fatigue after low-dose (initially 2 x 5 mg per day) administration of amifampridine. Patients 2-5 with PCS but no previous neurological disease also experienced an improvement in fatigue after treatment with amifampridine. In all five patients, the unphysiologically increased need for sleep was reduced with a simultaneous increase in the Bell score. This effect was reproducible in a double-blind discontinuation trial in two patients. An effect of amifampridine on PCS-associated fatigue has not yet been published.

Fatigue is a concomitant phenomenon of internal, neurological, and oncological diseases. It is associated with a serious reduction in quality of life and a considerable reduction in earning capacity. The underlying biological mechanisms are unknown, although an abnormal or excessive autoimmune and inflammatory response may play an important role [1]. Autoimmunity, endothelial damage to the blood vessels, and the persistence of the virus probably also play a role [2].

LEMS is an autoimmune-mediated neurological disorder characterized by muscle fatigue, decreased tendon reflexes, and symptoms of cholinergic overactivity. The underlying pathophysiology is the antibody-mediated blockade of voltage-gated calcium channels (VGCC), which decreases the release of acetylcholine in the synaptic junction [10]. Amifampridine is singularly indicated as an orphan drug for LEMS, and the improvement of coincident fatigue has been described [11]. Fatigue also improves in patients with motor neuron diseases such as amyotrophic lateral sclerosis (ALS) [12].

An effect of amifampridine administered to the patients in this collective casuistry on fatigue in diseases other than LEMS or motor neuron diseases has not yet been proven, but it would be plausible. This is derived from the fact that amifampridine is chemically closely related to fampridine (4-aminopyridine), both substances belonging to the group of aminopyridines. Additionally, both substances have the same physiological effect as K+ channel blockers. Accordingly, fampridine is used as an antagonist of the voltage-dependent potassium channels (Kv channels) as a symptomatic therapy for various neurological diseases. The positive effects have been explained by the blockade of axonal Kv channels, which improves conduction along demyelinated axons. There are also indications that fampridine could have further effects beyond its symptomatic mode of action [13]. Favorable effects of fampridine on cognitive performance [14] and cognitive fatigue [15] in patients with multiple sclerosis suggest this. An improvement was also observed with regard to depression and quality of life [16]. Finally, gait function also improved in patients with multiple sclerosis [17].

In principle, the possible side effects of any drug therapy must also be taken into consideration. In a study of 665 patients with multiple sclerosis, three with LEMS, and one with Steinert's disease, the safety profile of amifampridine was evaluated in routine clinical practice. Overall, 18.2% of patients experienced adverse drug reactions (ADRs) while taking moderate doses of 20-30 mg daily or up to 80 mg daily in patients with LEMS for up to 51 months. Most adverse effects were mild to moderate and transient. The most frequently observed adverse effects were paresthesias. There were six serious adverse events in the form of two epileptic seizures, one severe left-sided paresthesia, two cardiovascular disorders, and one drug-induced hepatitis [18]. In view of the massive impairment caused by PCS, the adverse drug reactions appear to be justifiable if amifampridine proves to be effective against fatigue in further studies.

## Conclusions

The present case collection provides strong evidence for the efficacy of amifampridine in PCS-associated fatigue. The efficacy of amifampridine would also be plausible, as the chemically closely related fampridine has already been shown to be effective in the treatment of fatigue in multiple sclerosis. For absolute proof, a randomized double-blind study is necessary, which is already planned. If proven, an extension of the indication of amifampridine for the treatment of PCS-associated fatigue would be urgent. Millions of people with PCS, for whom there is currently no adequate therapy, could then be effectively helped. As the author's (T.B.) LEMS disease is already at an advanced stage, it would be his most urgent wish to live to see this happen.
